# The severity of NEC is ameliorated by prostaglandin E2 through regulating intestinal microcirculation

**DOI:** 10.1038/s41598-023-39251-x

**Published:** 2023-08-17

**Authors:** Dandan Mo, Chun Deng, Bailin Chen, Xionghui Ding, Qin Deng, Hongjie Guo, Gongli Chen, Cuilian Ye, Chunbao Guo

**Affiliations:** 1 Department of Pediatrics, Yongchuan Hospital of Chongqing Medical University, 439 Xuanhua Rd, Chongqing, 402160 People’s Republic of China; 2 Department of General Surgery, Children’s Hospital of Chongqing Medical University, 20 Jinyu Ave., Chongqing, 400014 People’s Republic of China; 3 Department of Burn, Children’s Hospital of Chongqing Medical University, 20 Jinyu Ave., Chongqing, 400014 People’s Republic of China; 4 Department of Nutrition, Children’s Hospital of Chongqing Medical University, 20 Jinyu Ave., Chongqing, 400014 People’s Republic of China; 5 Department of Anesthesiology, Children’s Hospital of Chongqing Medical University, 20 Jinyu Ave., Chongqing, 400014 People’s Republic of China; 6 Department of Pediatric Surgery, Chongqing Health Center for Women and Children, Women and Children’s Hospital of Chongqing Medical University, 120 Longshan Rd., Chongqing, 401147 People’s Republic of China; 7 Department of Pediatrics, Women and Children’s Hospital of Chongqing Medical University, 120 Longshan Rd., Chongqing, 401147 People’s Republic of China; 8 School of Pharmacy and Bioengineering, Chongqing University of Technology, 69 Hongguang Ave., Chongqing, 400054 People’s Republic of China

**Keywords:** Physiology, Climate sciences, Medical research, Molecular medicine

## Abstract

Prostaglandin E2 (PGE2) is implicated in intestinal inflammation and intestinal blood flow regulation with a paradoxical effect on the pathogenesis of necrotizing enterocolitis (NEC), which is not yet well understood. In the current study, we found that PGE2, EP4, and COX-2 varied at different distances from the most damaged area in the terminal ileum obtained from human infants with NEC. PGE2 administration alleviated the phenotype of experimental NEC and the intestinal microvascular features in experimental NEC, but this phenomenon was inhibited by eNOS depletion, suggesting that PGE2 promoted intestinal microcirculatory perfusion through eNOS. Furthermore, PGE2 administration increased the VEGF content in MIMECs under TNFα stress and promoted MIMEC proliferation. This response to PGE2 was involved in eNOS phosphorylation and nitric oxide (NO) production and was blocked by the EP4 antagonist in vitro, suggesting that targeting the PGE2–EP4–eNOS axis might be a potential clinical and therapeutic strategy for NEC treatment. The study is reported in accordance with ARRIVE guidelines (https://arriveguidelines.org).

## Introduction

Necrotizing enterocolitis (NEC) is an emergency disease of the premature intestine that affects approximately 7% of preterm babies weighing between 500 and 1500 g^1^. Based on current evidence, the initial pathogenesis of NEC is unknown in preterm infants, and decreased splanchnic perfusion and microcirculation of the small intestine could be a result of subsequent intestinal tissue injury^2,3^. Following dysfunction of the intestinal microvasculature, bacterial translocation through the epithelial barrier exaggerates the proinflammatory response, which is associated with hypoxic damage during NEC development^4,5^. Therefore, intestinal blood flow modulation should offer a better direction toward novel treatments in NEC.

As the primary prostanoid in the intestine, prostaglandin E2 (PGE2) exhibits paradoxical effects^6–8^. Appropriate PGE2 plays a key role in intestinal homeostasis, especially in modulating blood flow through action at its receptors, which can modulate intestinal perfusion^9,10^. However, high levels of PGE2 are implicated in intestinal inflammation, leading to intestinal barrier breakdown in the pathogenesis of NEC as well as inflammatory bowel disease^7,11,12^. EP4, the endothelial PGE2 receptor, has previously been reported to be vital in myocardial ischemia/reperfusion injury^13,14^. Direct evidence about PGE2 in NEC pathogenesis has yet to be investigated due to the role of endothelial EP4 in intestinal microcirculation regulation.

In the present research, we found that PGE2 ameliorated the NEC phenotype through intestinal perfusion improvement by the eNOS phosphorylation signal modulated by the EP4 receptor. These findings support the notion that PGE2 could be a novel therapeutic target for NEC management.

## Materials and methods

### Human samples

Excised human specimens during the operation, including jejunums and ileums, were obtained following informed consent from all involved parents of the neonates in the Women and Children’s Hospital of Chongqing Medical University in 2020 with approval by the Institutional Review Board of Chongqing Medical University l from July 1, 2020, to July 30, 2022 (IRB, No.: WCHMU2021-052) in accordance with the relevant guidelines and regulations. Written informed consent was obtained for the participants' parent/legal guardian to participate in the study. The human samples included necrotizing intestinal samples from NEC patients (2 mm^3^, n = 8) resected during emergency laparotomy. Normal small intestine control samples (2 mm^3^, n = 6) were collected from infants undergoing surgery for ileal atresia, intestinal intussusception, Hirschsprung’s disease and imperforate anus. The demographics and characteristics of the controls are listed in Table 1. All samples were subjected to the following assessments: Western blotting, ELISA, and immunofluorescence evaluation. The study is reported in accordance with ARRIVE guidelines (https://arriveguidelines.org).

**Table 1:** Demographics and characteristics of Control.

No	Sex	Age (days)	Diseases	Site of collection
1	F	3	Imperforate anus	Colon
2	M	12	Ileal atresia	Jejunum
3	M	5	Duodenal septum	Ileum
4	F	7	Ileal atresia	Ileum
5	M	16	Ileal atresia	Jejunum
6	M	25	Duodenal septum	Jejunum

*F* female, *M* male.

### Experimental NEC models and drug interventions

The animal experimental protocols conformed to the guidelines for laboratory animal management (NIH publication No. 85–23) and were reviewed and approved by the animal care and use committee at the Women and Children’s Hospital of Chongqing Medical University, approval number (WCHMU2019-038). eNOS knockout (KO) mice (eNOS−/−, C57BL/6 J background) were kindly gifted by Prof. Jingyu Li from Sichuan University. Wild-type C57BL/6 mice were purchased from the Experimental Animal Center at Chongqing Medical University (Dr. Wenli Han). The previously described NEC stress protocol (formula gavage, hypothermia and hypoxia) was applied to five-day-old mouse pups^14,15^. Briefly, the pups were subjected to formula gavage every 4 h with 75 mL of puppy canine milk replacer (Pet-Ag, Hampshire, USA) and 15 g of Similac 60/40 (Ross Pediatrics, Columbus, OH, USA). Pups staying with the dam without any other treatment were used as controls. The experimental pups were observed, and clinical manifestations were recorded, including abdominal distension, apnea, rectal bleeding, body weight, feeding volume and mortality, in the following experimental period. Experimental NEC was evaluated by macroscopic bowel examination and microscopic assessment of the morphological and histological performance of the terminal ileum. The degree of mucosal injury of the terminal ileum was checked and scored based on a previously described histological scoring protocol^4,5^. The NEC diagnosis was verified by histological examination. The pups were euthanized using CO_2_ for intestinal tract harvest 3–5 days after NEC diagnosis. Part of the terminal ileum was subjected to the appropriate subsequent assessments listed below, such as quantitative real-time PCR (qPCR) and Western blotting.

**Figure 1:**
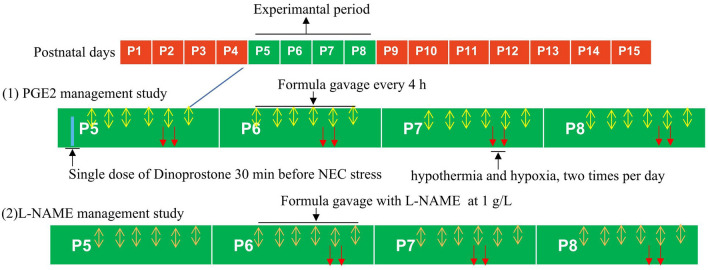
Flow diagram for the experimental NEC intervention.

In the PGE2 management study, litters of newborn pups were intraperitoneally injected with either a single dose of dinoprostone (prostaglandin E2, Sigma Aldrich, 300 mg/kg body weight) or vehicle control (0.9% sodium chloride) at an equivalent volume 30 min before NEC stress. In the L-NAME (N-nitro-L-arginine methyl ester hydrochloride, Sigma–Aldrich) management study, L-NAME was dissolved in the formula gavage at a concentration of 1 g/L and administered every 4 h. The flow diagram of experimental management is presented in Fig. 1.

### Preparation of intestinal microvascular endothelial cells

Intestinal microvascular endothelial cells (MIMECs) were isolated and cultured as reported previously^4,5^. When the MIMECs were cultured for 7 days, we obtained almost pure cultures of MIMECs (90% confluence). The MIMECs were subjected to the preliminary dosage selection assay, and the current dosage of TNF-α (30 ng/mL) was selected for 12 h for subsequent experiments. For pharmacological PGE2 management and inhibition of various PGE2 receptors, the culture system was further cultured with the following reagents and final concentrations: stabilized PGE2 analog 16,16-dimethyl PGE2 (dmPGE2, 1 μM, R&D Systems), EP1 inhibitor (10 μM, SC 51322; R&D Systems), EP2 inhibitor (10 μM, PF 04418948; R&D Systems), EP3 inhibitor (10 μM, L-798,106; R&D Systems), and EP4 inhibitor (10 μM, L-161,982, R&D Systems).

### ELISA analysis of VEGF

MIMECs were cultured in 24-well plates at 90% confluence. Before PGE2 management, the serum was deprived for 24 h of starvation. The culture supernatant was collected for VEGF concentration measurement using enzyme-linked immunoassay (ELISA) kits (R&D Systems) following the manufacturer’s instructions.

### Nitric oxide (NO) assessments

Isolated intestinal segments were homogenized and centrifuged to remove cell debris, and the supernatant was saved for NO analysis. The NO concentrations in the intestinal samples were determined using the modified Griess reaction and the total NO assay kit (Beyotime Institute of Biotechnology, China) as described previously^4,5^. NOS inhibition was performed by incubation with L-NAME (500 mM, Sigma‒Aldrich).

### Cell proliferation detection

A 3-(4,5-dimethylthiazol-2-yl)-2,5-diphenyl tetrazolium bromide (MTT) assay was conducted to assess cell proliferation. MIMECs were seeded in a 96-well plate (500 cells/well) for 24 h, and TNFα (30 ng/mL) was administered for 12 h in RPMI-1640 medium (Gibco, Australia). MTT solution (20 μL; 5 mg/mL in PBS, pH 7.4; Solarbio Life Sciences, Peking, China) and dimethyl sulfoxide (DMSO) (150 μL; Sigma) were then successively incubated within each well with constant shaking. A Thermo Fisher Scientific microplate reader was utilized for the optical absorbance measurement at 490 nm.

### PGE2 concentration measurement

MIMEC lysates and terminal ileum samples were collected and homogenized for protein concentration measurement. Samples were diluted 1:10 with assay buffer before analysis. The concentration of PGE2 in ileal tissue homogenates was determined utilizing a high-sensitivity PGE2 enzyme immunoassay kit (Assay Designs, Ann Arbor, MI, USA) following the manufacturer’s instructions.

### Quantitative real-time polymerase chain reaction (RT‒PCR)

MIMEC lysates and terminal ileum segments were collected and subjected to RNA extraction with TRIzol (Invitrogen, Carlsbad, CA, USA). Reverse transcription was then conducted for cDNA using a reverse transcription kit (Roche, Germany). RT‒PCR was performed using a Roche LightCycler 480 real-time PCR instrument (Roche, Germany). The primer sequences for EP1, EP2, EP3, EP4, and eNOS are presented in Table 2. The mRNA expression levels of EP1, EP2, EP3, EP4, IL-6, TNFα, CD31, VEGFR2 and eNOS were normalized to 18S rRNA expression. The annealing temperature was 59 °C for all genes examined.

**Table 2:** The primer sequences for the real-time PCR measurement.

Gene	Forward (5′-3′)	Reverse (5′-3′)
Human EP1	TTCGGCCTCCACCTTCTTTG	CACCAACACCAGCATTGGGCT
Human EP2	GCTCCTTGCCTTTCACGATTT	AGGATGGCAAAGACCCAAGG
Human EP3	GTCGTGTACCTGTCCAAGCA	GTCGTGTACCTGTCCAAGCA
Human EP4	AATTCGTCCGCCTCCTTGAG	CACCACCCCGAAGATGAACA
Human β-actin	CTCCATCCTGGCCTCGCTGT	GCTGTCACCTTCACCGTTCC
Mouse eNOS	AGGACATATGTTTGTCTGCGGCGA	AAATGTCCTCGTGGTAGCGTTGCT
Mouse IL-6	GGCTAAGGACCAAGACCATCCAA	TCTGACCACAGTGAGGAATGTCCA
Mouse TNFα	CATCTTCTCAAAATTCGAGTGACAA	TGGGAGTAGACAAGGTACAACCC
Mouse CD31	AGCTAGCAAGAAGCAGGAAGGACA	TAAGGTGGCGATGACCACTCCAAT
Mouse VEGFR2	CTGGAGCCTACAAGTGCTCG	GAGGTTTGAAATCGACCCTCG
Mouse β-actin	CCCTGGAGAAGAGCTACGAG	CGTACAGGTCTTTGCGGATG

### Intestinal SIgA and β-defensin-2 measurement

PBS containing 0.02% sodium azide was subjected to passage through the intestinal lumen, and then intestinal mucus was obtained. The washout mixture was prepared as previously described (n = 6 in each group)^3^. The levels of secretory immunoglobulin A (SIgA) and β-defensin-2 in the intestinal mucus were determined using ELISA kits (USCN, China) according to the manufacturer's instructions.

### Western blot analysis

The intestinal samples were processed as previously reported. The total protein concentration was quantified using the Bradford protein assay. The different samples (30 μg protein) were first subjected to electrophoresis and then transferred onto polyvinylidene fluoride (PVDF) membranes for primary antibody incubation. The primary antibodies utilized in the current research included p-eNOSSer1177 (Cell Signaling Technology, USA), eNOS (Cell Signaling Technology), CD31 (Cell Signaling Technology), VEGFR2 (Cell Signaling Technology) and GAPDH (Santa Cruz Biotechnology). The appropriate secondary antibodies were incubated to visualize the immunoreactive bands. β-actin (Sigma) was used as a loading control. The relative intensities of the target bands were measured utilizing Kodak 1D 3.5.4 software (Kodak Scientific Imaging System, Rockville, MD, USA).

### Confocal immunofluorescence assays

The slides in different groups were first subjected to incubation with the appropriate primary antibodies, including anti-CD31 (1:50, Sigma) and anti-BrdU (1:50) and then incubated with the appropriate secondary antibodies. DAPI (4’,6-diamidino-2-phenylindole; 1:1,000, Sigma) was used to counterstain the nuclei. Finally, the slides were mounted and analyzed under a fluorescence confocal microscope (Leica Microsystems Heidelberg GmbH, Heidelberg, Germany). Quantitative analysis was performed using the plugin ‘colocalization threshold’ of WCIF ImageJ software (National Institutes of Health, Bethesda, MD, USA) by at least two independent investigators. We selected representative sections in 5 fields for each segment of the intestine (magnification, 200 ×) from 6 mice under each experimental condition.

### Bacterial translocation by microbiological culture of blood

Blood from experimental pups was collected in a sterile fashion and plated onto blood agar plates (containing 5% sheep blood) to assess total aerobic bacteria or on BBL Brucella agar plates (containing 5% horse blood; BD) as described previously^4,5^. The colony-forming units (CFUs) were counted following incubation in aerobic or anaerobic conditions at 37 °C for 48–72 h.

### Statistical analysis

GraphPad Prism (version 6) (GraphPad Software, Inc., La Jolla, CA, USA) was used for data analysis. Continuous parametric data are presented as the mean ± SEM for normal distribution and were analyzed using ANOVA. The survival curve was generated with Kaplan‒Meier analysis following the log-rank (Mantel‒Cox) test. P values < 0.05 were considered statistically significant.

### Ethics approval

All experiments were approved by the animal care and use committee of Chongqing Medical University.

## Results

### Variation in intestinal PGE2 in human infants

**Figure 2:**
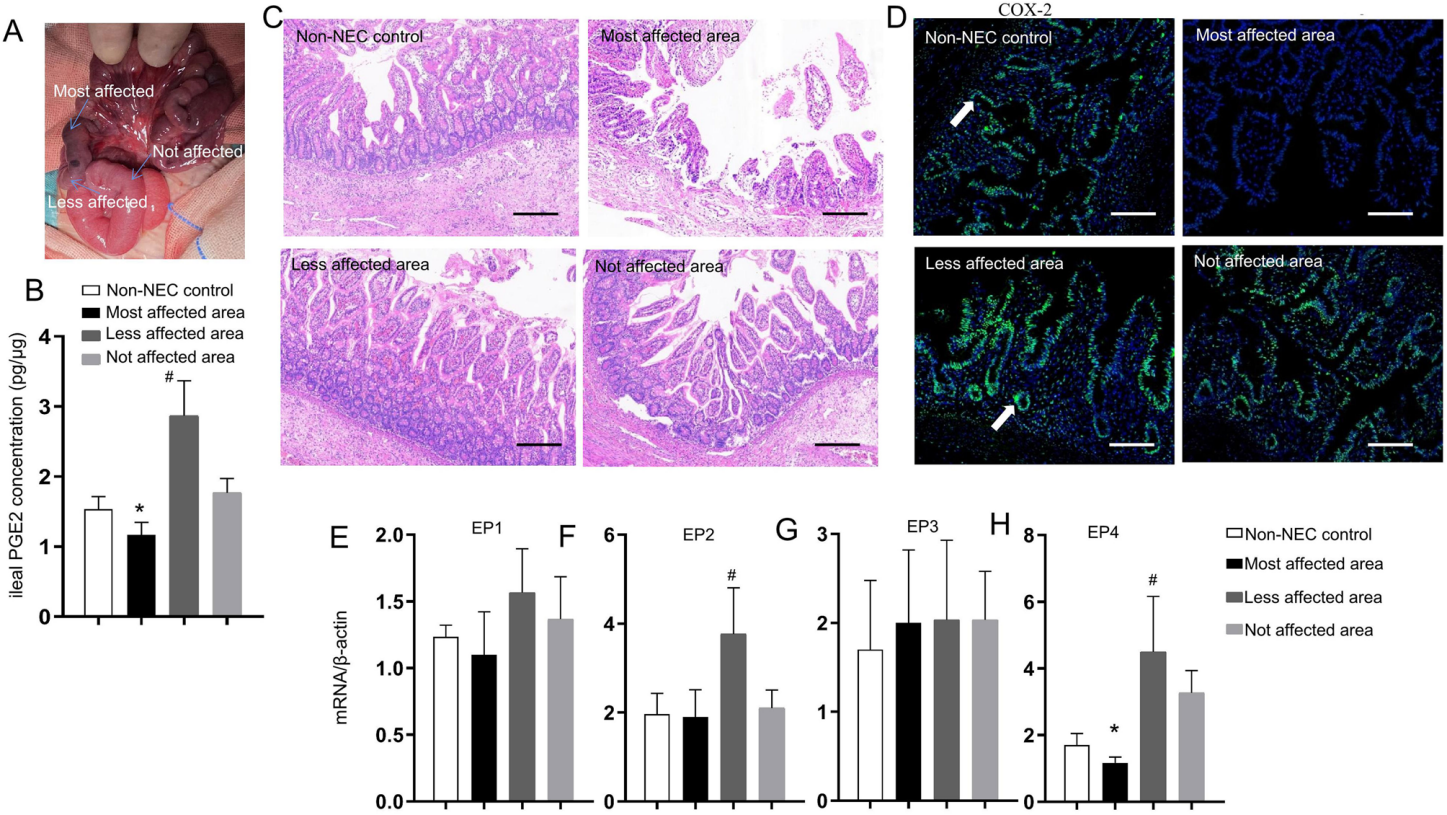
Involvement of PGE2 in NEC in human intestinal tissue. (**A**) The typical features of assessed samples within the jejunum and ileum segments; (**B**) PGE2 levels were measured in ileal homogenates from human patients. PGE2 levels in intestinal tissue lysates calculated per µg of protein. *P < 0.01, ^#^P < 0.01 vs. non-NEC control; (**C**) Representative morphology of the human ileum (the area of necrosis and perforation, less affected ileum, nonaffected ileum and no-NEC control) using hematoxylin and eosin staining. Scale bars: 100 µm; (**D**) Representative immunofluorescence staining for COX-2 in the terminal ileum of NEC patients and non-NEC control patients (white arrows). Scale bars: 100 µm. Three independent experiments were repeated with similar results. The mRNA expression levels of EP1 (**E**), EP2 (**F**), EP3 (**G**), and EP4 (**H**) relative to β-actin mRNA were assessed in intestinal tissues using qRT‒PCR. Values represent means ± SEMs. *P < 0.01; ^#^P < 0.01 (one-way ANOVA).

We first compared the PGE2 value in terminal ileal homogenates at varying distances from the most affected field, which were resected from infants suffering from NEC. The gross manifestations of the samples assessed are presented in Fig. 2A. The median PGE2 concentration value was lowest or even absent in the most damaged intestinal area of NEC, whereas the median PGE2 concentration value was significantly higher at the less involved terminal ileum than in the other positions of intestinal tissues and non-NEC controls (Fig. 2B). The serum value of PGE2 did not exhibit a difference between the NEC patients and non-NEC controls (data not shown).

The severity of mucosal injury was maximal in the most damaged intestinal segment based on the extent of submucosal edema, epithelial sloughing, neutrophil infiltration, and derangement of intestinal villi, which gradually exhibited a better performance away from the affected fields, even resembling the characteristics of non-NEC control infants (Fig. 2C). Positive immunoblotting for COX-2 in the terminal ileum was distributed diffusely with brown granules in the villi and were reduced or even absent at the site of most damaged areas compared with the less affected and non-NEC control ileum (Fig. 2D). Arrows indicate COX-2-positive cells. The mRNA levels of four EP receptors (EP1–4) were assessed in the different areas of the terminal ileum by means of RT–PCR; the EP2 and EP4 mRNA levels were observed to be increased at the less involved terminal ileum than in the other positions of intestinal tissues and non-NEC controls (Fig. 2E–H).

### Effects of PGE2 on NEC severity

**Figure 3:**
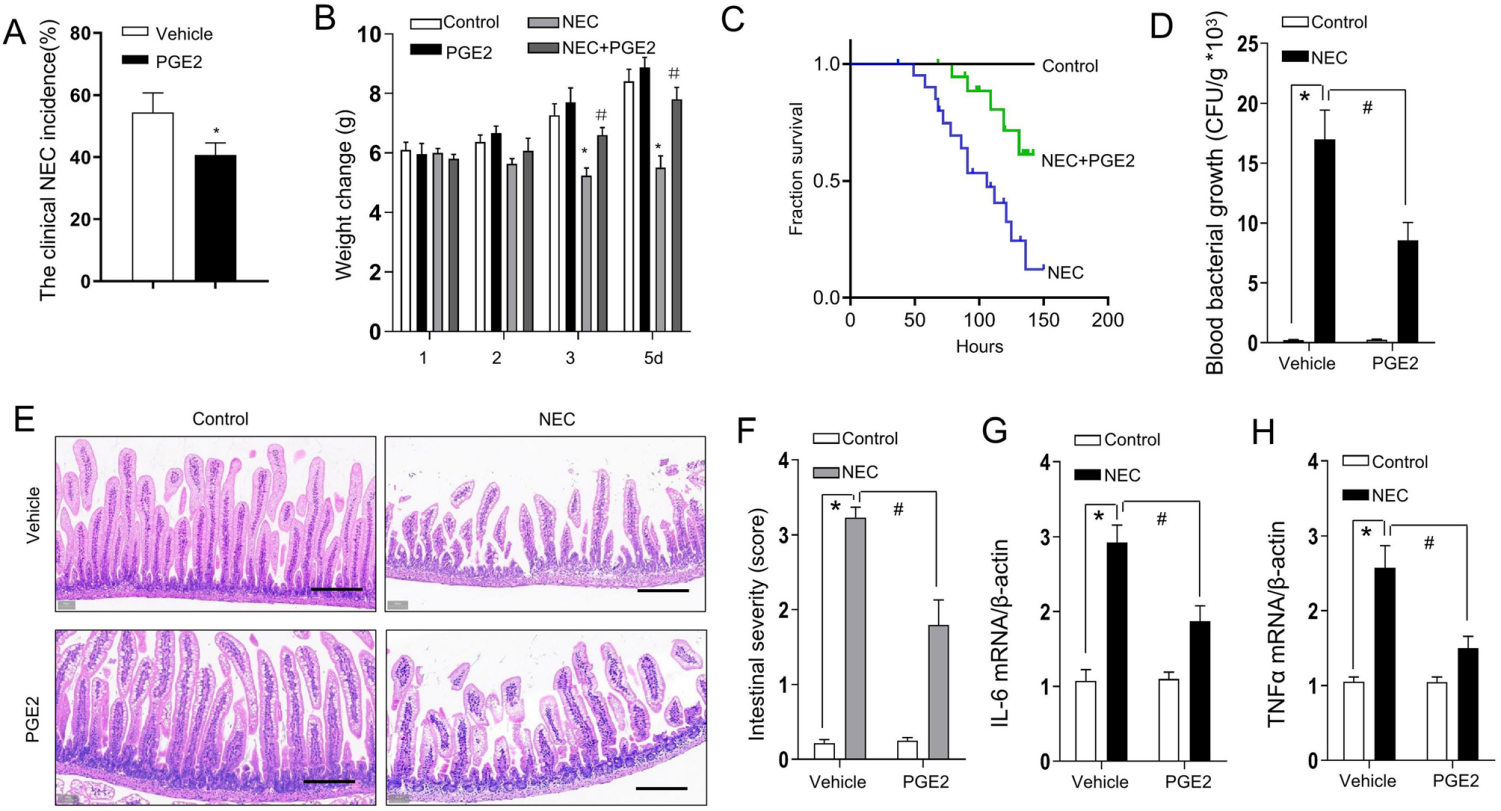
PGE2 administration attenuated the severity of experimental NEC. (**A**) PGE2 administration reduced the incidence of experimental NEC (damage scores over 2) in pups under hypothermic and hypoxic conditions. Column values represent the average of multiple experiments (n = 10–12 mice per group). Bars, SEM *P < 0.01 (one-way ANOVA); (**B**) Weight change of established NEC treatments as indicated. Columns, the average values of multiple experiments (n = 10–12 mice per group); bars, SEM, *P < 0.01, vs. control; ^#^P < 0.01 (one-way ANOVA); (**C**) Kaplan‒Meier analysis for the pups among the different managements. Data represent percent survival (n = 10–18 mice per group); (**D**) Quantification of blood bacterial growth from pups managed as indicated. Values represent means ± SEMs. *p < 0.01 vs. the corresponding WT control; ^#^P < 0.01 vs. the corresponding WT mice with NEC (one-way ANOVA); (**E**) Representative terminal ileums with H&E staining for the pups from three independent replicate coverslips with experimental NEC treated as indicated. Scale bars: 100 μm; (**F**) The severity scores for NEC were measured by a pathologist (n = 10–18 mice per group) according to morphological performance (**E**). Columns, means; bars, SEM. *P < 0.01; ^#^P < 0.01 (one-way ANOVA). The mRNA levels of IL-6 (**G**) and TNFα (**H**) in the intestinal tissues were measured from the pups treated as indicated (n = 7). Data represent the mean ± SEM. *P < 0.01; ^#^P < 0.01 (one-way ANOVA).

We next investigated the effect of PGE2 on the phenotype of NEC. PGE2 decreased the NEC incidence of pups stressed with formula gavage and hypoxia from approximately 55% in the control to 40% in PGE2 administration (Fig. 3A). A slight loss of body weight was observed for pups with established NEC compared with the control pups. PGE2 administration ameliorated the weight loss of pups after the onset of NEC and resulted in a healthier appearance (Fig. 3B). Furthermore, PGE2 administration promoted the survival of the NEC pup model (Fig. 3C). We further evaluated whether PGE2 administration affected the microbial translocation from the intestine to the blood circulation extent of bacterial translocation using the standard blood culture method and found that PGE2 suppressed bacterial translocation across the intestinal barrier, as promoted by formula feeding and hypoxia (Fig. 3D). The histological features of the established model of NEC were characterized by incomplete mucosal epithelial cells and microvillus integrity as well as high inflammatory cell infiltration (Fig. 3E). PGE2 administration ameliorated the histological appearance above, including inflammatory cell infiltration and intestinal architecture. Accordingly, the pathology scores were reduced with PGE2 administration (Fig. 3F) compared with their counterparts. To evaluate the effects of PGE2 administration on proinflammatory cytokine levels, we detected the expression of IL-6 and TNFα. As indicated in Fig. 3G and H, PGE2 administration suppressed the levels of IL-6 and TNFα, suggesting that detrimental proinflammatory injury during the pathogenesis of NEC could be ameliorated by PGE2.

### PGE2 accelerated eNOS phosphorylation at Ser1177 in experimental NEC

**Figure 4:**
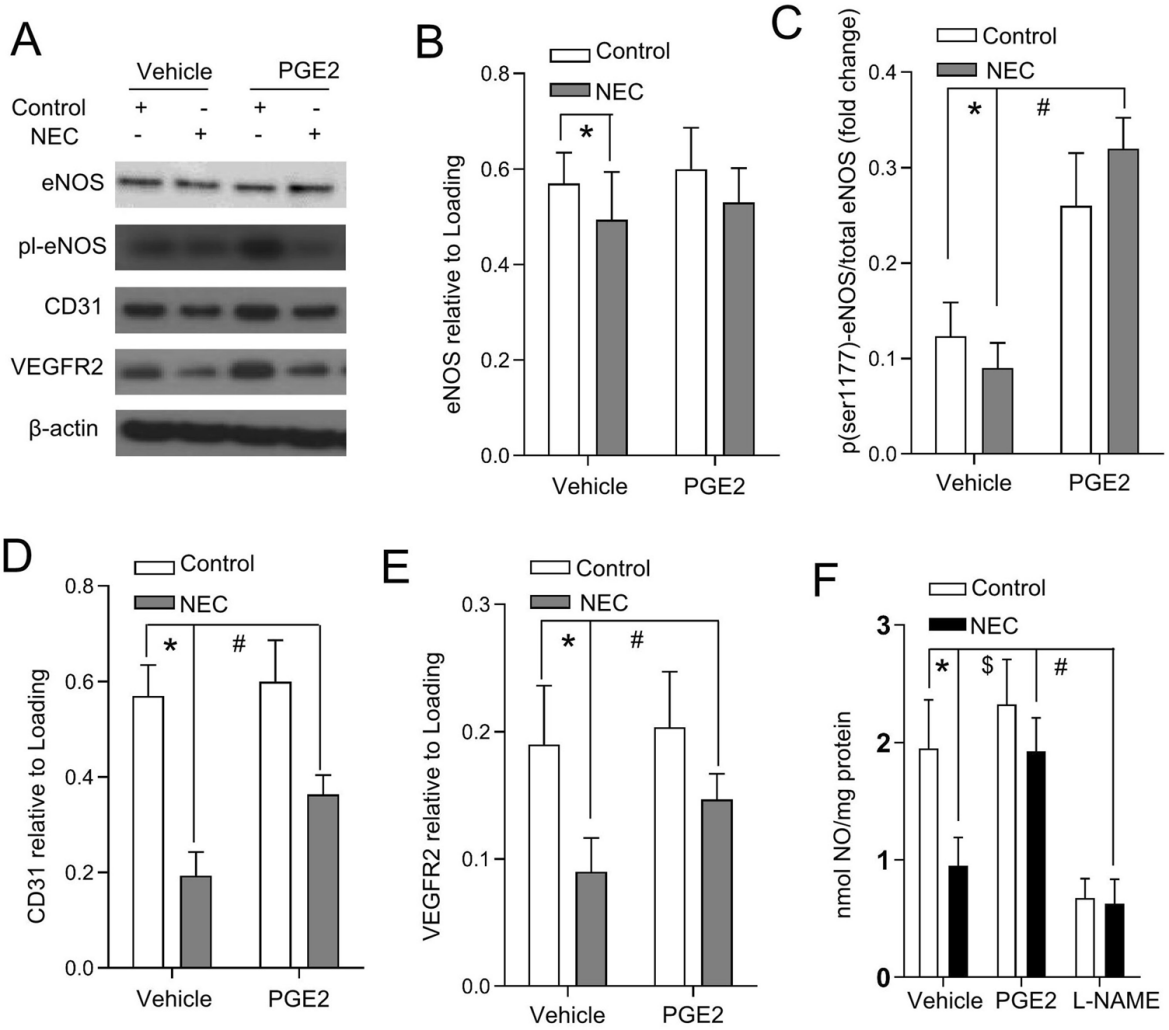
Effect of PGE2 on eNOS phosphorylation in experimental NEC pups. (**A**) Western blot analysis of total eNOS, phosphorylated eNOS, CD31 and VEGFR2 expression in intestinal homogenates of the pups treated as indicated. The representative bands represent blots of a minimum of three independent experiments. Quantitative analysis of total eNOS (**B**), the phosphorylated eNOS/eNOS ratio (**C**), CD31 (**D**) and VEGFR2 (**E**) are presented as indicated in triplicate. The histograms are represented as the means ± SEMs. *P < 0.01; ^+#^P < 0.01 (one-way ANOVA); (**F**) Nitric oxide production was assessed in the intestinal segments of the indicated groups. The eNOS inhibitor L-NAME was used to test the eNOS-dependent mechanism during NO measurement. Columns: means; bars, SEM; *P < 0.01; ^$^P < 0.01, and ^#^P < 0.01 (one-way ANOVA).

eNOS can be activated to produce NO by phosphorylation at the Ser1177 site (p-eNOSSer1177). To examine whether eNOS activity acted as a signal transmitter of PGE2 in NEC-associated pathogenesis in vivo, we next assessed total eNOS and p-eNOSSer1177 in experimental NEC. Formula feeding and hypoxia exposure decreased the total and p-eNOSSer1177 levels of intestinal eNOS expression in the intestine (Fig. 4A–C). PGE2 administration increased the activity of eNOS, as confirmed by the significantly upregulated phosphorylation of eNOS at Ser1177 (Fig. 4A–C) in the intestine. Importantly, PGE2 administration did not affect the transcription of eNOS (Fig. 4E), suggesting that PGE2 increases eNOS activity largely through eNOS phosphorylation. Significant reductions in CD31 and VEGFR2 protein levels were present in pups under NEC stress, whereas PGE2 administration reversed both the levels of CD31 and VEGFR2 (Fig. 4A,D,E).

We further evaluated whether PGE2 signaling was essential for NO production, which was associated with the activation of eNOS activity. Consistent with the p-eNOSSer1177 level, a reduction in NO production following NEC was present in the intestinal segments. NO production exhibited an approximately 50% increase when experimental NEC pups were subjected to PGE2 administration (Fig. 4F). L-NAME restrained NO production by approximately 70–80%, indicating that NO production in the intestine is eNOS dependent. The results support the notion that PGE2 could activate eNOS phosphorylation and regulate NO production.

### The effect of PGE2 on NEC requires eNOS

**Figure 5:**
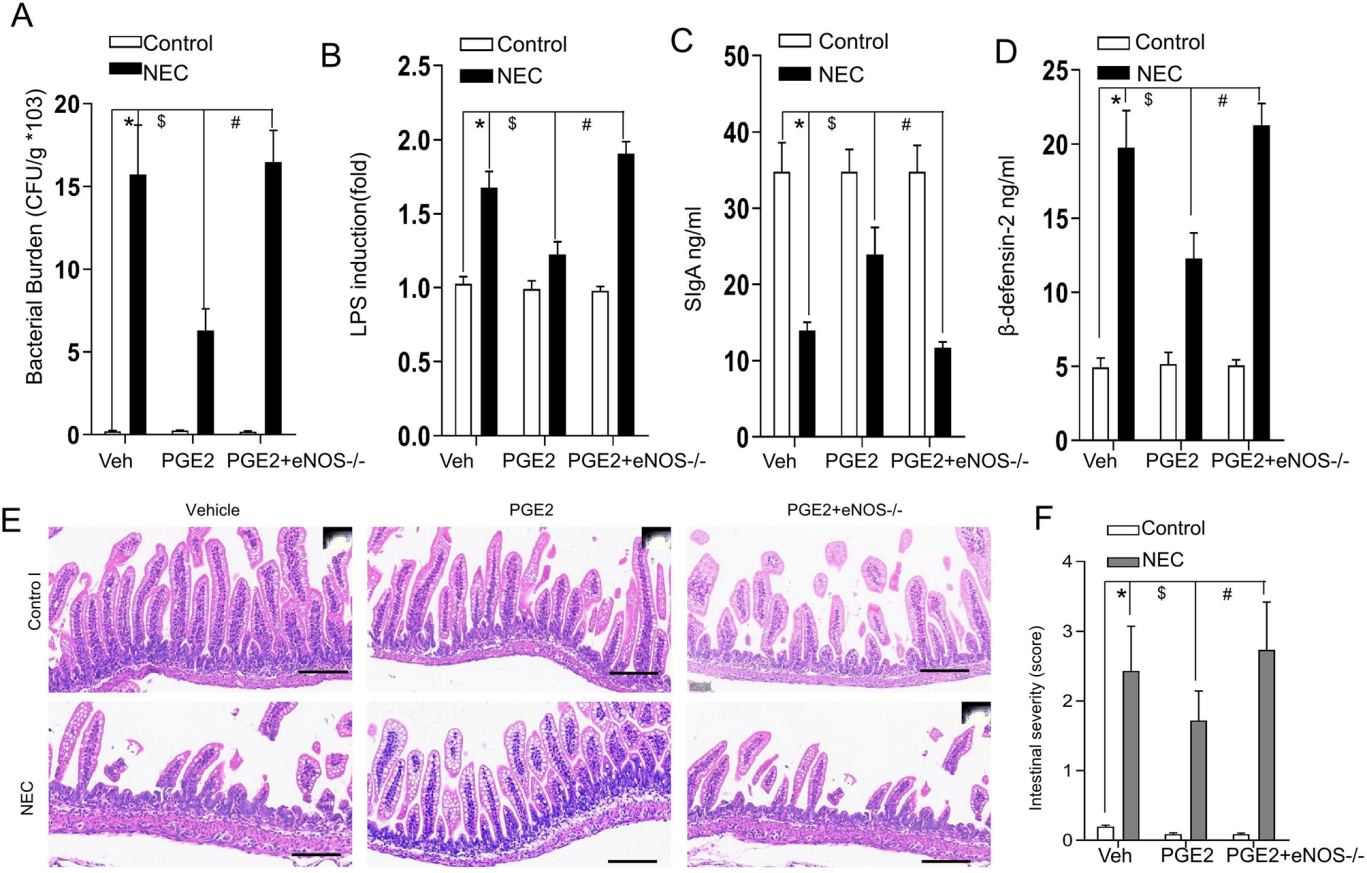
eNOS depletion abolished the effect of PGE2 on NEC. (**A**) Blood bacterial growth quantification under the indicated conditions (n = 5–10 animals per group). (**B**) Endotoxin levels (LPS) are presented relative to control-fed animals (n = 5–10 animals per group). The concentrations of SIgA (**C**) and β-defensin-2 (**D**) were measured in the terminal ileum of mice treated as indicated (n = 5–10 animals per group). The histograms are represented as the means ± SEMs. *P < 0.01 vs. WT control; ^#,$^P < 0.01 vs. the corresponding WT mice with NEC; ^§^P < 0.01 vs. the corresponding eNOS−/− mice with NEC (one-way ANOVA); (**E**) Representative terminal ileums with H&E staining for the pups from three independent replicate coverslips with experimental NEC treated as indicated. Scale bars: 100 μm; (**F**) NEC severity scores based on morphological performances were measured by the pathologist (n = 10–18 mice per group). Columns, means; bars, SEM. *P < 0.01 vs. the corresponding WT control; ^#,$^P < 0.01 vs. the corresponding WT mice with NEC; ^§^P < 0.01 vs. the corresponding eNOS−/− mice with NEC (one-way ANOVA).

We have previously reported that eNOS is involved in the pathogenesis of NEC. We therefore administered PGE2 to eNOS−/− mice in the experimental NEC model and explored bacterial translocation through LPS measurement, a bacterial translocation marker, and direct bacterial culture. As illustrated in Fig. 5A and B, the bacterial burden and LPS levels were significantly increased in experimental NEC, and PGE2 administration abolished this bacterial translocation. PGE2 had no effect on the bacterial burden, LPS production, SIgA or β-defensin-2 when administered to eNOS−/− mice (Fig. 5A–D), suggesting that the mechanistic insights of eNOS account for the protective effects of PGE2.

Moreover, PGE2 did not relieve the disease severity of NEC in mice with eNOS deficiency, as proven by the gross and histological appearance of the involved intestine (Fig. 5E,F). These findings suggested that PGE2 attenuated the severity of NEC via eNOS as an intermediate signal.

### PGE2 rescued intestinal microcirculation injury during NEC development

**Figure 6:**
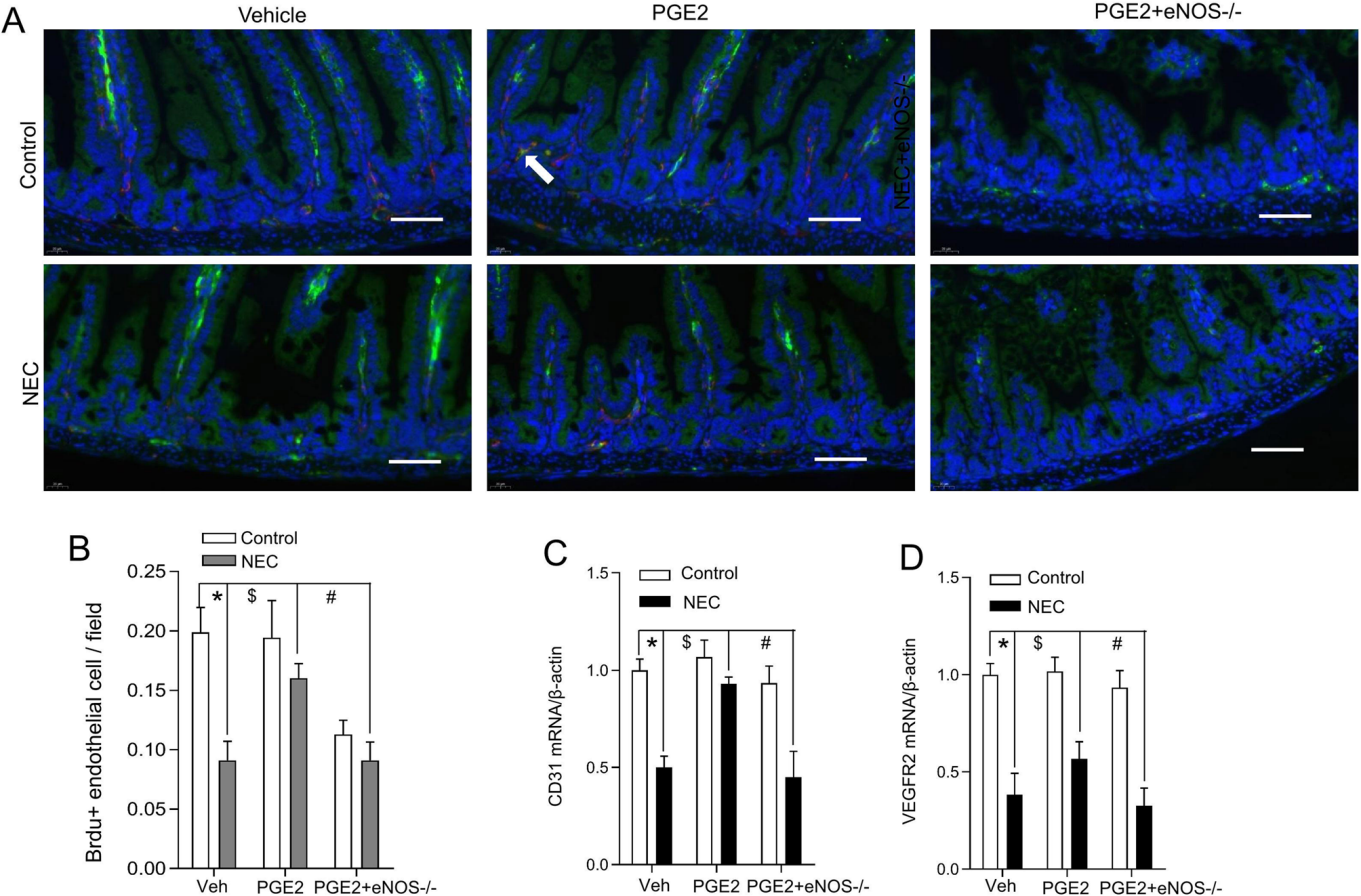
PGE2 promotes intestinal endothelial cell proliferation. (**A**) Confocal staining of BrdU (red‒purple) and CD31 (green) was performed to trace endothelial cell proliferation in ileum villi samples from pups treated as indicated. Representative immunofluorescent images are presented. Scale bar, 50 μm; (**B**) Quantitative endothelial cell measurement based on confocal immunofluorescence staining (5–8 fields/sample, n = 5/group). Columns: means; bars, SEM; *P < 0.01; ^$^P < 0.01; ^#^P < 0.01 (one-way ANOVA). The mRNA expression of CD31 (**C**) and VEGFR2 (**D**) with respect to β-actin was assessed in at least three independent experiments in intestinal homogenates from the indicated treatment groups (n = 6). Columns: means; bars, SEM; *P < 0.01; ^#^P < 0.01 (one-way ANOVA).

PGE2 has previously been suggested to promote endothelial cell proliferation in the intestine, which is associated with the pathogenesis of NEC. We further tracked endothelial cell proliferation within the intestinal villi in experimental NEC using BrdU and the endothelial cell marker CD31. As assessed in the representative confocal images (Fig. 6A), NEC stress reduced the colocalization of BrdU and CD31 (yellow/orange, arrow), but PGE2 administration mitigated this trend in pups with experimental NEC, illustrating the importance of PGE2 for endothelial cell proliferation. Notably, PGE2 did not alleviate the changes in pups with eNOS deficiency due to intestinal colocalization of BrdU and CD31 (Fig. 6B,C), indicating the critical role of eNOS signaling in regulating intestinal microcirculation in PGE2 management. We further compared the expression of the CD31 and VEGFR2 genes using quantitative RT‒PCR. As shown in Fig. 6C and D, the expression levels of these angiogenesis factors were significantly downregulated in the NEC group compared with the control group (Fig. 1D). PGE2 promoted their expression during NEC development, and eNOS depletion abolished its effects.

### PGE2 promotes the activity of microvascular endothelial cells via the EP4 receptor

**Figure 7:**
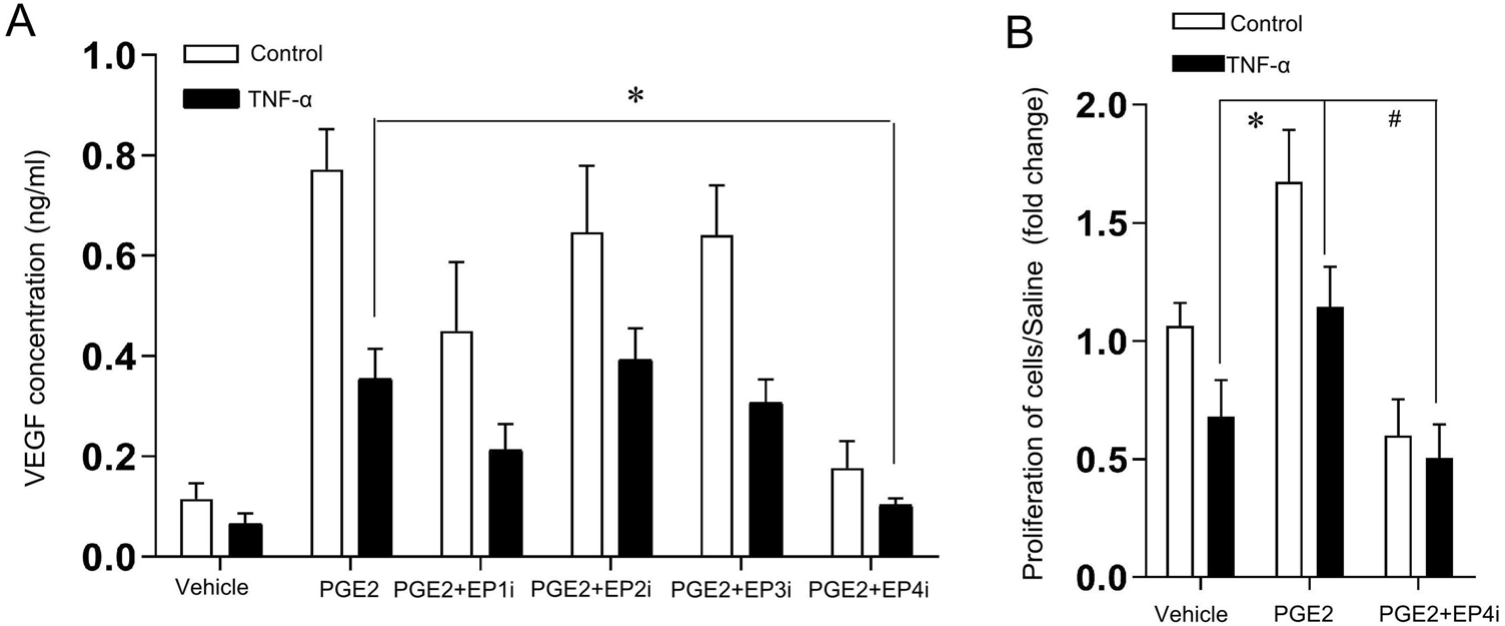
In vitro assessments of endothelial cell viability modulated by the PGE2 pathway. (**A**) VEGF levels in the conditioned media of MIMECs were determined by ELISA. MIMECs were treated as indicated, including PGE2, PGE2 and specific pharmacological inhibitors of EP1 (EP1i, SC 51,322), EP2 (EP2i, PF 04,418,948), EP3 (EP3i, L-798, 106), or EP4 (EP4i, L-161, 982). Columns, means; bars, SEM. *P < 0.01 (one-way ANOVA). (**B**) Cell proliferation was assessed in MIMECs treated as indicated (n = 6). Columns: means of at least three independent repeat experiments; bars, SEM; *P < 0.01; ^#^P < 0.01 (one-way ANOVA).

Next, we determined the amount of VEGF secreted by the isolated MIMECs in response to PGE2 using ELISA. The fresh supernatant for endothelial cell culture contained approximately 0.1 ng/ml VEGF, which was increased after PGE2 administration for 24 h (Fig. 7A). PGE2 can signal through four PGE receptors (EP1, EP2, EP3, and EP4) in the small intestine and colon. Next, to elucidate the molecular mechanism by which receptors mediate PGE2 function, we measured the VEGF level in isolated MIMECs under various specific pharmacological inhibitors to inhibit the four receptors, including inhibitors of EP1 (SC 51322), EP2 (PF 04418948), EP3 (L-798,106), and EP4 (L-161,982). Among them, PGE2-induced VEGF production could be blocked by only an EP4 inhibitor (L-161,982) (Fig. 7B), confirming the mechanistic involvement of EP4 signaling in PGE2-induced VEGF production (Fig. 7B). We also explored the effect of PGE2 on cell proliferation in MIMECs under TNF-α exposure. Compared with the vehicle, TNF-α restrained the proliferation of MIMEC (P < 0.05), and PGE2 promoted MIMEC proliferation, whereas an EP4 inhibitor (L-161,982) further restrained PGE2-associated MIMEC proliferation under TNF-α exposure.

### PGE2 increases eNOS activity in intestinal microvascular endothelial cells via the EP4 receptor

**Figure 8:**
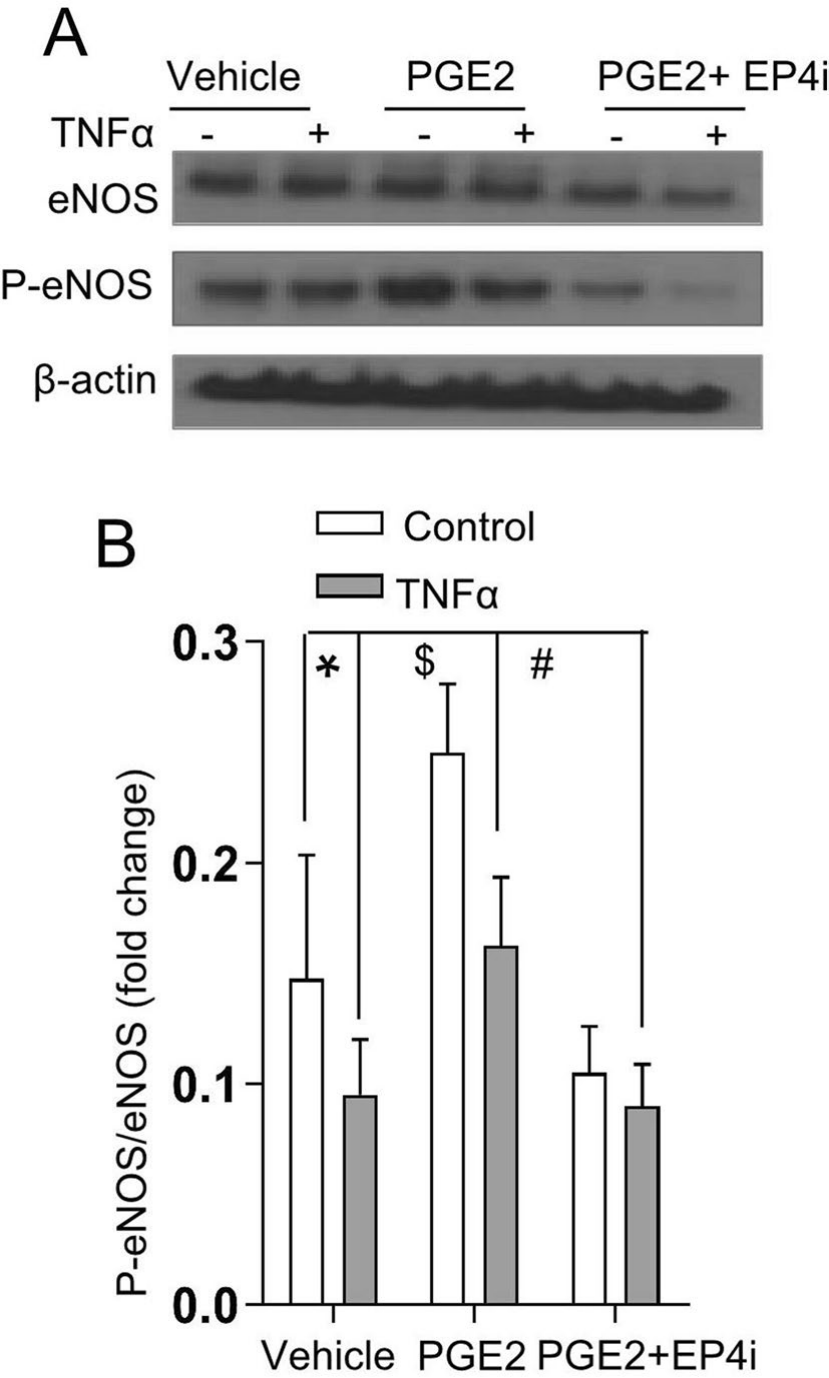
PGE2 modulates eNOS phosphorylation through the EP4 receptor in MIMECs. (**A**) Western blot analysis was performed to explore total eNOS and phosphorylated proteins in MIMECs treated as indicated. The blots are representative Western blots of at least three independent experiments. Quantitative total eNOS and phosphorylation expression data (**B**) were calculated regarding the corresponding loading control (n = 3). The data are presented as the means ± SEMs; *P < 0.01; ^$^P < 0.01; ^#^P < 0.01 (one-way ANOVA).

We further assessed the function of eNOS, which is involved in microvascular endothelial cell viability, under various interventions. We demonstrated that TNF-α prevented eNOS phosphorylation at Ser1177 (p-eNOSSer1177) and that PGE2 abrogated the suppressive effect of TNF-α (Fig. 8A,B). Importantly, the EP4 inhibitor disrupted this effect of PGE2 on eNOS phosphorylation (Fig. 8A,B), indicating the involvement of EP4 in PGE2-eNOS signaling.

## Discussion

In the present study, we explored the functional activity of PGE2 in the intestinal microcirculation in the pathogenesis of NEC and explored its therapeutic potential. We found that PGE2 and its EP4 receptor are essential in the modulation of the neonatal intestinal microcirculation and are associated with the successful relief of the severity of experimental NEC. We further clarified that PGE2 modulated eNOS activation and NO production through EP4 signaling, an essential signaling pathway in the intestinal microcirculation mechanism during NEC development.

In previous research, intestinal barrier function was shown to be impaired in NEC, with changes in mucosal permeability and vulnerability to bacterial invasion, which likely account for intestinal microvascular and inflammatory injury^4,5^. Here, we indeed detected constriction of intestinal microvascular perfusion in pups with experimental NEC. Indeed, in the current research, the most affected terminal ileum had severe damage in the top of the villi, which should be the site of necrosis and perforation, whereas in the less affected ileum, there should be the site of most severe intestinal inflammation. We hypothesized that to suppress intestinal inflammation, the PEG2 level is compromised. Naturally, the ileum farther away from this less affected area regained a normal PEG2 level identical to that of non-NEC control neonates. PGE2 has been identified as the predominant beneficial vasodilatory gut prostaglandin^15^. As an essential prostaglandin synthesis enzyme, COX-2 blockade with indomethacin could inhibit subsequent prostaglandins, which may ultimately contribute to the intestinal injury observed in clinical patients^16,17^. Interestingly, administration of PGE2 (dinoprostone) protected against vasoconstriction in concert with NSAIDs^22^. The PGE2 content confers a protective effect in experimental scenarios and is beneficial for various biological processes^18–21^. Similar to a previous report, we demonstrated that PGE2 administration not only significantly increased intestinal microvascular perfusion in NEC pups but also relieved the severity of intestinal damage and contributed to survival in experimental NEC. A recent report in mice also showed that an increase in intestinal blood flow was observed with PGE2 and EP4 agonist administration, supporting the notion that the PGE2/EP4 signaling pathway is essential for neonatal intestinal microvascular perfusion^23^. It is unknown which cells are responsible for PGE2 production in the intestine, although many different cell types may produce prostanoids. Our immunofluorescence microscopy experiments clearly indicated the epithelium as the main area where the majority of COX-2 was located. The crucial prostaglandin synthesis enzyme is COX-2, which accounts for the rate-limiting step of prostaglandin production^24,25^. These observations suggested that PGE2 should be produced in the epithelium of the intestine.

Depending on the availability of EP receptors, the role of PGE2 in intestinal homeostasis is paradoxical as either a vasoconstrictor or vasodilator. High levels of COX-2 and PGE2 are detrimental to the intestine through inflammatory damage, while low levels are critical to maintaining intestinal barrier integrity^26,27^. Prior ex vivo pharmacological studies demonstrated that PGE_2_ administration increased arteriolar and/or capillary reperfusion through EP2 and/or EP4 receptor signaling^28^, suggesting that as vasorelaxant receptors, EP2 and/or EP4 receptor signaling may be protective. The EP4 receptor is identified as the primary mediator of prostaglandin-induced vasodilation in intestinal perfusion, which is different from the other 3 EP receptors^6^. Here, in a model of NEC, we confirmed that EP4 is a protective prostaglandin receptor that reduces intestinal microvascular injury. Of course, PGE2 and its receptor EP4 have been confirmed to be involved in the development of NEC due to the vasodilatory effect of endothelial EP4^29,30^, although cardiac and renal function changes have not been observed to be associated with endothelial EP4. Conventional EP4 knockout mice have been shown to have perinatal lethality because of patent ductus arteriosus. Furthermore, EP4 depletion models are more susceptible to colitis with immune downregulation and disruption of mucosal barrier function^31^. Recently, antagonists selective for EP2 and EP4 receptors have been developed and utilized to confirm the findings generated from knockout mice^32^. In our current investigation, the EP4 antagonist abrogated the effects of PGE2 in terms of intestinal microvascular perfusion, suggesting a protective function of EP4 in intestinal microvascular injury to improve microcirculation functional recovery after NEC, indicating the beneficial role of PGE2/EP4 signaling, which should represent an attractive therapeutic target for NEC management. Previous research also demonstrated that EP4 antagonist administration decreased intestinal blood flow, supporting our current findings^23^.

We previously indicated that eNOS-mediated vasodilation within the intestinal endothelium contributes to intestinal microcirculation perfusion through NO production, leading to the alleviation of NEC^4,5^. Impaired NO production and dysfunction of eNOS activity in endothelial cells result in relaxation of smooth muscle cells and an increase in blood flow, which should account for the vasoprotective function during NEC development^33,34^. Our findings demonstrate that endothelial EP4 promotes eNOS activity and subsequent NO production, which is essential for NEC development. eNOS capacity has been well documented to be activated by phosphorylating the Ser1177 residue (p-eNOSSer1177) or inactivated through Thr495 (p-eNOSThr495) residue phosphorylation^35,36^. In the current investigation, we demonstrated that vascular p-eNOSSer1177 was profoundly inhibited following hypothermia/hypoxia or TNFα stress in cultured MIMECs and that this effect could be reversed by PGE2 administration. We further demonstrated a high phosphorylation level of eNOS at the Ser1177 residue (p-eNOSSer1177) (which leads to the activation of eNOS activity) in endothelial cells following PGE2 administration in pups with NEC, verifying the histochemical findings and corroborating the important contribution of PGE2 via eNOS activation. Additionally, increased p-eNOSSer1177 in isolated MIMECs was observed in response to PGE2 administration or EP4 activation. Conversely, inhibition of EP4 eliminated the phospho-eNOS elicited by PGE2 administration, suggesting that PGE2 can promote p-eNOSSer1177 and NO production via EP4 signaling. Consistent with this, PGE2 promoted microvascular endothelial cell viability and proliferation, highlighting the critical role of PGE2-EP4-p-eNOSSer1177 in the pathogenesis of NEC through endothelial proliferation.

## Conclusion

Taken together, we have uncovered an essential role of endothelial PGE2-EP4 in eNOS activation for NO production during NEC pathogenesis and discovered that activation of PGE2 or EP4 could be a new therapeutic target that may rescue endothelial dysfunction through eNOS activation (Supplementary Figures).

### Supplementary Information

Supplementary Figures.

## Data Availability

The data that support the findings of this study are available from the corresponding author upon reasonable request.

## Abbreviations

COX-2: Cyclooxygenase-2
MIMECs: Intestinal microvascular endothelial cells
NEC: Necrotizing enterocolitis
NO: Nitric oxide
PGE2: Prostaglandin E2
TNF-α: Tumor necrosis factor-α
VEGF: Vascular endothelial growth factor
