# Supplementary figures and images for: The severity of NEC is ameliorated by prostaglandin E2 through regulating intestinal microcirculation
Source: Sci Rep. 2023 Aug 17;13:13395. doi: 10.1038/s41598-023-39251-x (PMC10435505; doi:10.1038/s41598-023-39251-x)

A

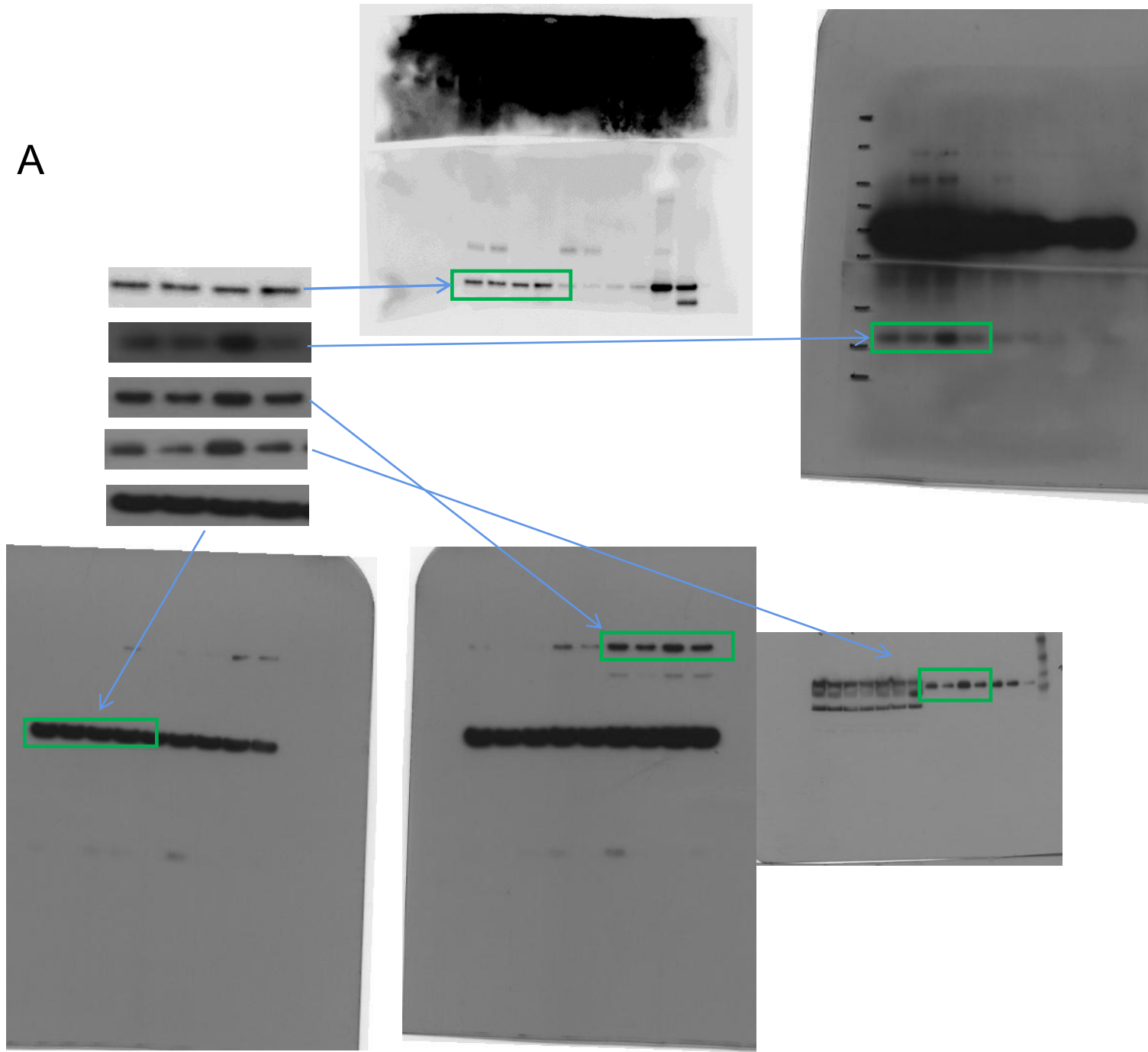

A

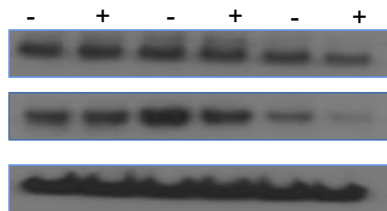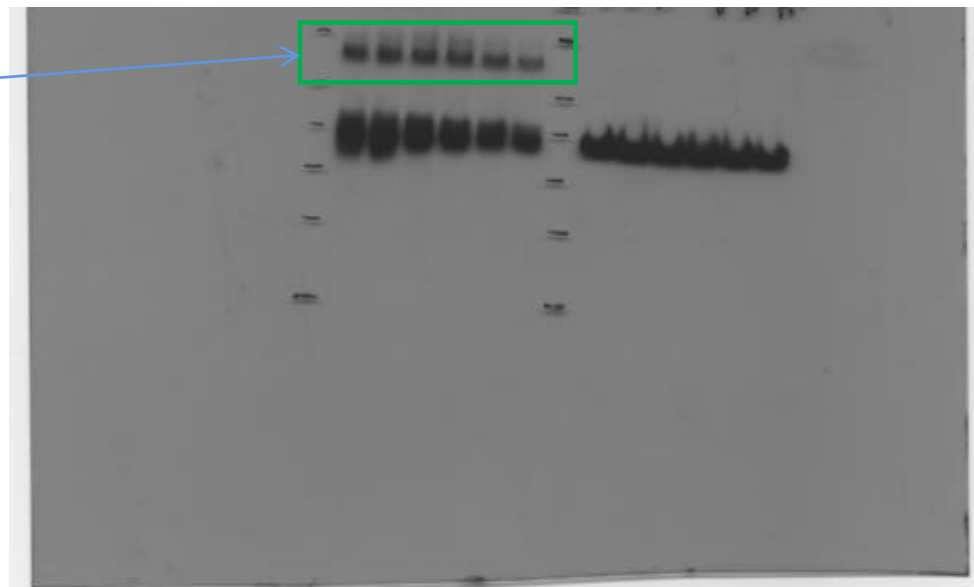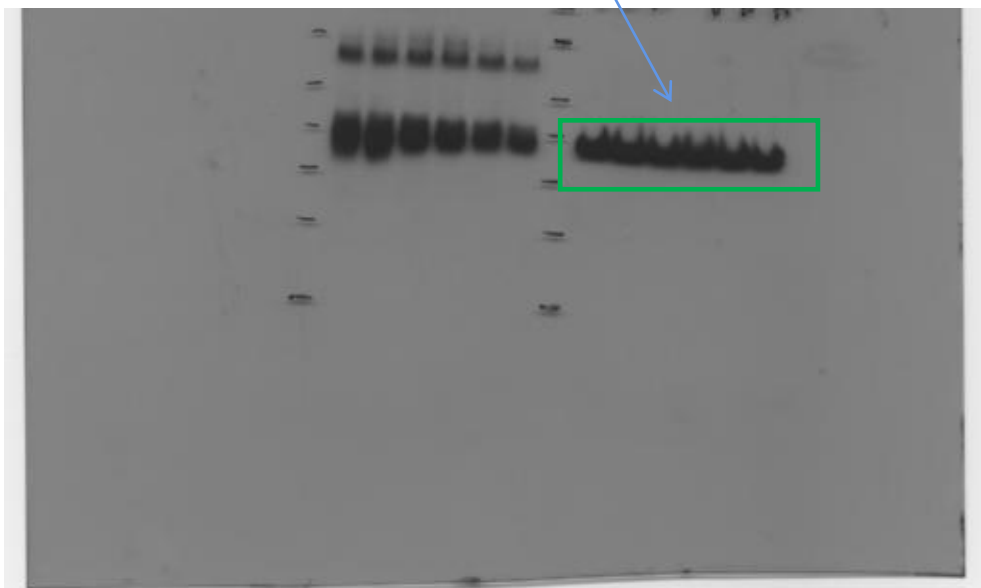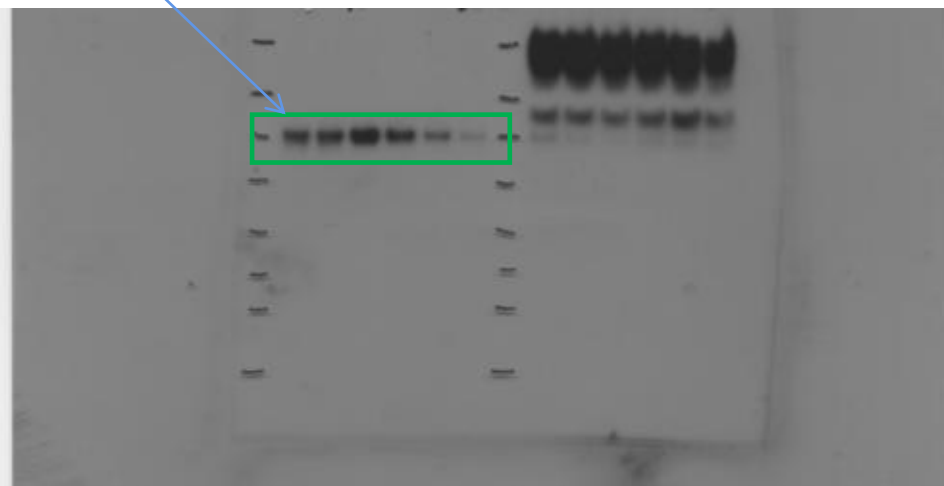

Supplement: Supplementary file 1 — Supplementary Figures. [file 41598_2023_39251_MOESM1_ESM.pdf]
